# NF-κB/miR-223-3p/ARID1A axis is involved in *Helicobacter pylori* CagA-induced gastric carcinogenesis and progression

**DOI:** 10.1038/s41419-017-0020-9

**Published:** 2018-01-09

**Authors:** Fenghua Yang, Yugang Xu, Chao Liu, Cunying Ma, Shuiyan Zou, Xia Xu, Jihui Jia, Zhifang Liu

**Affiliations:** 1 Department of Biochemistry and Molecular Biology, School of Basic Medicine, Shandong University, Jinan, China; 2 Department of General Surgery, Center Hospital of Taian City, Taian, China; 3 Department of Minimal Access Surgery of Tumor, Center Hospital of Taian City, Taian, China; 4 Department of Microbiology, Key Laboratory for Experimental Teratology of Chinese Ministry of Education, School of Basic Medicine, Shandong University, Jinan, China

## Abstract

Infection with *Helicobacter pylori* (*H. pylori*) and the resulting gastric inflammation is regarded as the strongest risk factor for gastric carcinogenesis and progression. NF-κB plays an important role in linking *H. pylori-*mediated inflammation to cancer. However, the underlying mechanisms are poorly understood. In this study, we find that *H. pylori* infection induces miR-223-3p expression in * H. pylori* CagA-dependent manner. NF-κB stimulates miR-223-3p expression via directly binding to the promoter of miR-223-3p and is required for *H. pylori* CagA-mediated upregulation of miR-223-3p. miR-223-3p promotes the proliferation and migration of gastric cancer cells by directly targeting ARID1A and decreasing its expression. Furthermore, miR-223-3p/ARID1A axis is involved in CagA-induced cell proliferation and migration. In the clinical setting, the level of miR-223-3p is upregulated, while ARID1A is downregulated significantly in human gastric cancer tissues compared with the corresponding noncancerous tissues. The expression level of miR-223-3p is significantly higher in *H. pylori*-positive gastric cancer tissues than that in *H. pylori-*negative tissues. Moreover, a negative correlation between miR-223-3p and ARID1A expression is found in the gastric cancer tissues. Taken together, our findings suggested NF-κB/miR-223-3p/ARID1A axis may link the process of *H. pylori*-induced chronic inflammation to gastric cancer, thereby providing a new insight into the mechanism underlying *H. pylori*-associated gastric diseases.

## Introduction

Gastric cancer is one of the most common malignancies in the world and ranks third in terms of cancer-related death among all cancers^1^. *H. pylori* infection causes chronic gastritis and peptic ulcer, and is considered to be the strongest risk factor for the development of gastric cancer^2,3^. *H. pylori* is a gram-negative bacterium and usually colonizes on the human gastric mucosa^4^. Persistent infection with *H. pylori* may cause immunological response and chronic inflammatory reaction which is a crucial step in the initiation and development of gastric cancer^5^. The pathogenicity of *H. pylori* is attributed largely to its various virulence components and the most extensively studied *H. pylori* virulence factor is CagA^6^. The CagA protein, a 120–140 kDa protein encoded by the cag pathogenicity island (*cagPAI*), can be injected into gastric epithelial cells via a type IV secretion system (T4SS) and behaves as a bacterial oncoprotein^7^. Infection with CagA-positive *H. pylori* strains is associated with higher grades of gastric inflammation and an increased risk for gastric cancer compared with infection with CagA-negative strains, thereby highlighting the important role for CagA in *H. pylori*-associated gastric diseases^8,9^. Within gastric epithelial cells, CagA activates multiple critical pathways, such as nuclear factor (NF)-κB, β-catenin, phosphatidylinositol-3-kinase/AKT and Src/MEK/extracellular signal-regulated kinase pathway^10–13^, and causes a series of cellular events, finally leading to the malignant transformation of gastric epithelial cells. Among these pathways, the activation of NF-κB by CagA plays an important role in *H. pylori*-induced transformation from inflammation to cancer. NF-κB is a transcription factor that regulates the expression of anti-apoptotic and proliferation-associated genes. It activates different pro-inflammatory cytokines and chemokines and seems to be a key molecular link between inflammation and cancer^14^. However, the exact molecular mechanisms of CagA/NF-κB-dependent linkage between inflammation and cancer remain to be elucidated.

miRNAs, small non-coding RNAs of ~19–25 nucleotides in length^15^, play important roles in many biological processes including tumorigenesis and progression. miRNAs interact directly with specific target mRNAs and cause the translational repression or degradation of the target genes^16,17^. In human cancer, miRNAs can undergo aberrant regulation and function as oncogenes or tumor suppressor genes, depending on the regulated target genes^18^. Recent reports have demonstrated the regulatory role of miRNAs in *H. pylori*-induced inflammation and carcinogenesis. For example, Xiao et al.^19^ reported that *H. pylori* infection induced the up-regulation of miR-155 through NF-κB and AP-1 pathways, which, in turn, diminished the production of inflammatory cytokines via attenuating NF-κB activity. Zou et al.^20^ showed that *H. pylori* new toxin Tip-α activated NF-κB to promote inflammation and carcinogenesis by inhibiting miR-3178 expression in gastric mucosal epithelial cells. Matsushima et al.^21^. found 31 differentially expressed miRNAs by miRNA microarrays between the *H*. *pylori*-infected and -uninfected mucosa. Of these miRNAs, only has-miR-223-3p showed increased expression in *H. pylori*-positive mucosa, but the potential mechanism is not clear. In a previous study, the promoter region of miR-223-3p was reported to contain putative NF-κB binding site^22^. Therefore, we wonder whether miR-223-3p is involved in CagA-mediated inflammation to gastric cancer by serving as a downstream target of NF-κB.

In this study, we find that *H. pylori* CagA induces miR-223-3p expression through NF-κB pathway. Moreover, we validate the oncogenic role of miR-223-3p by repressing ARID1A (AT-rich interacting domain containing protein 1A) expression. Therefore, our findings suggest that NF-κB/miR-223-3p/ARID1A axis may link the process of *H. pylori*-induced chronic inflammation to gastric cancer and miR-223-3p may serve as a novel target for the intervention of the malignance.

## Results

### *H. pylori* induces miR-223-3p expression depending on CagA in gastric cancer cells

**Fig. 1:**
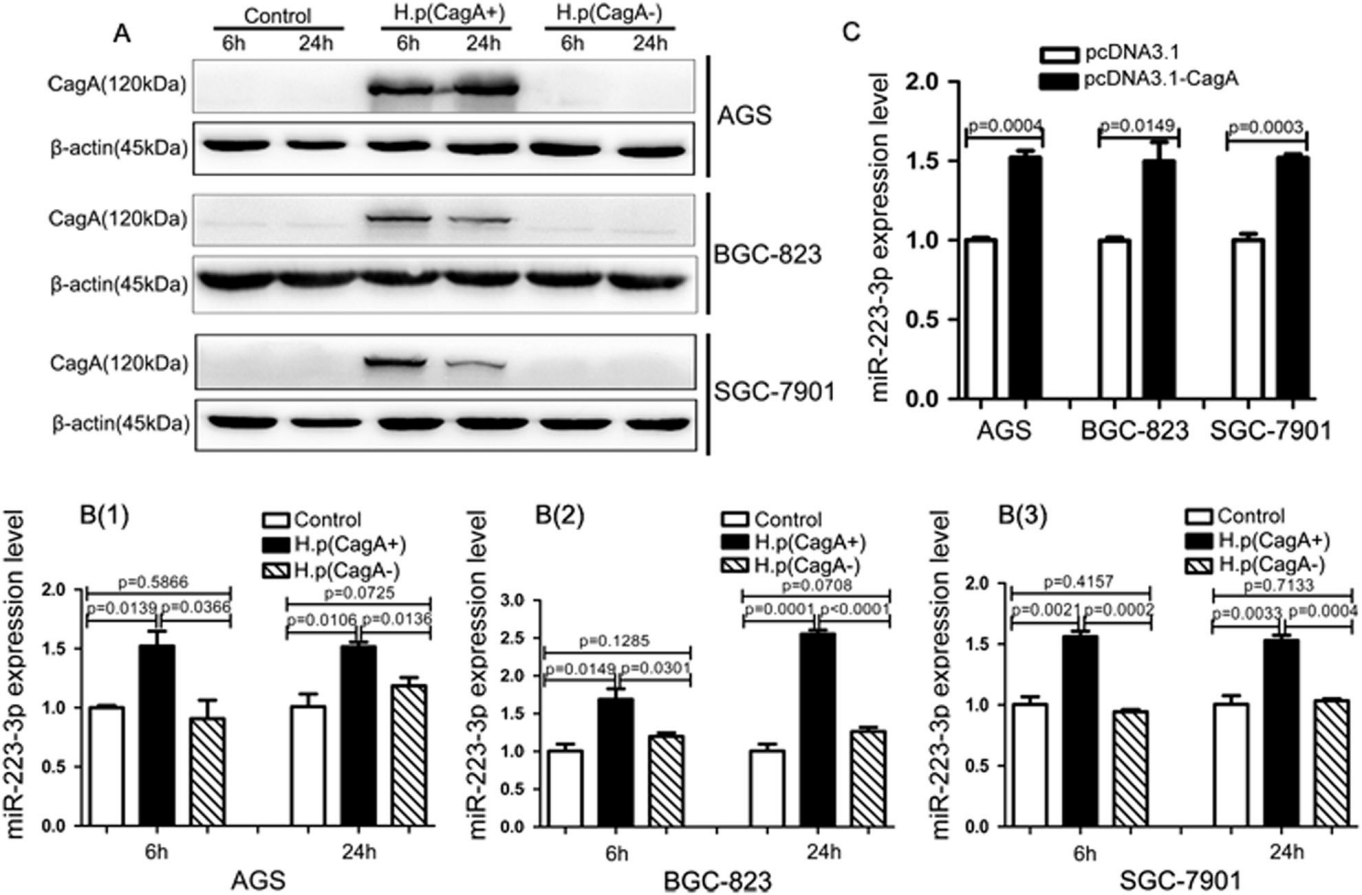
*H. pylori* induces miR-223-3p expression depending on CagA in gastric cancer cells **a** The expression of CagA was analyzed by western blot in AGS, BGC-823 and SGC-7901 cells infected with *H. pylori* (CagA^+^ or CagA^-^) at MOI (multiplicity of infection) of 100:1 for 6 or 24 h. **b** (1–3) qRT-PCR analysis of the expression of miR-223-3p in the gastric cancer cells infected with *H. pylori* (CagA^+^) or *H. pylori* (CagA^-^). Data are the means±SD of three independent experiments. **c** qRT-PCR analysis of the expression of miR-223-3p in AGS, BGC-823 and SGC-7901 cells transfected with control vector (pcDNA3.1) or CagA expression vector (pcDNA3.1-CagA). Data are the means±SD of three independent experiments

To investigate the regulatory role of *H. pylori* infection on miR-223-3p expression, we infected the gastric cancer cells AGS, BGC-823 and SGC-7901 with *H. pylori* 26695 (CagA^+^) for 6 and 24 h^3,23^ and determined the expression of miR-223-3p with quantitative real-time PCR (qRT-PCR). The results showed that the *H. pylori* CagA was expressed in *H. pylori* (CagA^+^)-infected cells (Fig.1a) and miR-223-3p expression level was significantly increased with *H. pylori* (CagA^+^) infection in all the three cells (Fig. 1b). In addition, we noticed that the expression of CagA protein was decreased at 24 h compared with 6 h in BGC-823 and SGC-7901 cells, while the decrease of CagA protein expression was deferred to 48 h in AGS cells (Fig. S1). We speculate that the downregulation of CagA protein expression in the cells is due to autophagy-mediated clearance of exogenous protein. Since different cells have different genetic backgrounds and biological characteristics, the time for the clearance is different.

To further determine whether *H. pylori* CagA was responsible for the increased expression of miR-223-3p, we used a isogenic 26695 CagA mutant strain (CagA^−^) to infect the cells and found that the isogenic 26695 CagA mutant strain infection had no effect on the expression of miR-223-3p (Fig. 1a, b). Furthermore, we used CagA expression vector (pcDNA3.1-CagA) to transfect the gastric cancer cells and found that miR-223-3p expression was significantly increased with pcDNA3.1-CagA transfection (Fig. 1c). Taken together, these results suggested that *H. pylori* infection induced miR-223-3p expression in CagA-dependent manner.

### NF-κB is required for the induction of miR-223-3p upon *H. pylori* stimulation

**Fig. 2:**
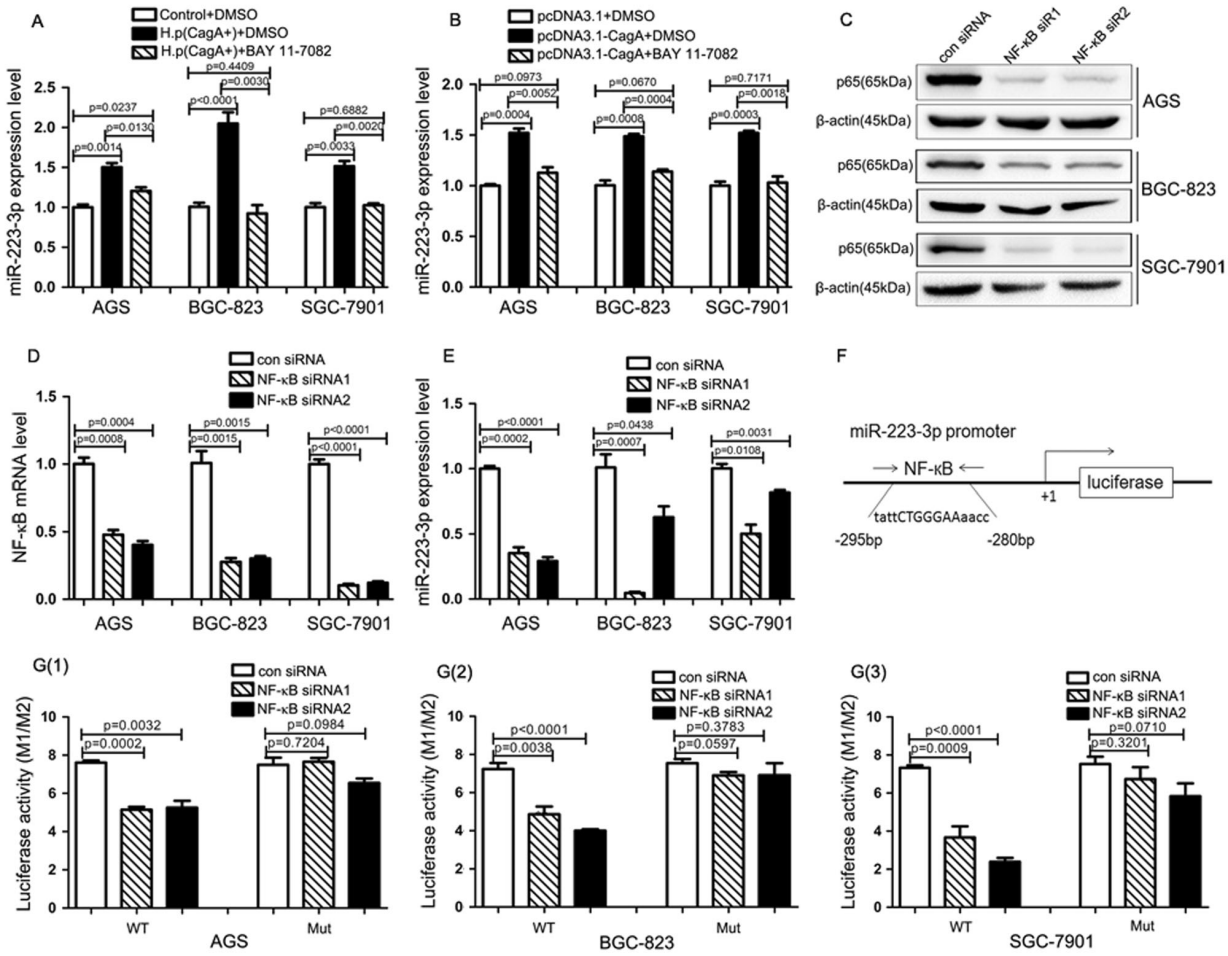
NF-κB binds to the miR-223-3p promoter and is involved in CagA-induced upregulation of miR-223-3p **a** miR-223-3p expression was analyzed by qRT-PCR in AGS, BGC-823 and SGC-7901 cells treated with *H.* *pylori* (CagA^+^) alone or together with NF-κB inhibitor BAY 11-7082. **b** qRT-PCR analysis of the expression of miR-223-3p in AGS, BGC-823 and SGC-7901 cells transfected with CagA expression vector (pcDNA3.1-CagA) alone or together with NF-κB inhibitor BAY 11-7082. **c** Western blot analysis of NF-κB protein level in AGS, BGC-823 and SGC-7901 cells transfected with control siRNA or NF-κB siRNAs. **d** qRT-PCR analysis of the NF-κB mRNA level in AGS, BGC-823 and SGC-7901 cells transfected with control siRNA or NF-κB siRNAs. **e** qRT-PCR analysis of the expression of miR-223-3p in the gastric cancer cells transfected with control siRNA or NF-κB siRNAs. **f** Schema of the putative NF-κB binding sites in the miR-223-3p promoter region. **g** (1–3). Wild-type (WT) or the mutant (Mut) pGL3-miR-223-3p promoter construct was co-transfected with control siRNA or NF-κB siRNAs into AGS, BGC-823 and SGC-7901, and the dual luciferase activity was determined at 48 h after transfection

It has been demonstrated that the *H. pylori* CagA-mediated malignant transformation of gastric epithelial cells are closely related to NF-κB activity, which is a key molecular link between inflammation and oncogenesis initiation and progression^24^. Therefore, we next determined whether NF-κB was involved in CagA-mediated miR-223-3p upregulation. We used the NF-κB pathway inhibitor BAY 11-7082 to treat the gastric cancer cells and then determined the expression of miR-223-3p. As shown in Fig. 2a, pretreatment of gastric cancer cells with BAY 11-7082 abrogated the upregulation of miR-223-3p induced by *H. pylori* (CagA^+^) infection. Similarly, BAY 11-7082 treatment also abrogated the upregulation of miR-223-3p mediated by pcDNA3.1-CagA transfection in AGS, BGC-823 and SGC-7901 cells (Fig. 2b). To further confirm the role of NF-κB, we transfected NF-κB-specific small interfering RNA (siRNA) into the gastric cancer cells to knock down NF-κB expression (Fig. 2c, d). qRT-PCR results showed that NF-κB siRNA reduced miR-223-3p expression level in the three cells (Fig. 2e).

As a transcription factor, NF-κB has been demonstrated to bind to the binding sites on the promoters of target genes and regulate their transcription. It has been reported that there is a putative NF-κB binding site in the promoter of miR-223-3p^22^. Therefore, we assume that NF-κB binds to the promoter of the primary miR-223-3p and regulates its transcription directly. In order to investigate the role of NF-κB signaling in the transcriptional regulation of the miR-223-3p, we constructed a wild-type luciferase reporter vector containing 1063 bp of the human pri-miR-223 5′ proximal genomic region and a mutant luciferase reporter vector in which the NF-κB binding sites were mutated (Fig. 2f). The AGS, BGC-823 and SGC-7901 cells were co-transfected with the luciferase reporter vector and NF-κB siRNA. The luciferase assay showed that inhibition of NF-κB expression reduced the luciferase activity driven by the wild-type pGL3-miR-223-3p promoter, whereas NF-κB siRNA has no significant effect on the mutant miR-223-3p promoter activity (Fig. 2g). These results suggested that NF-κB was involved in CagA-mediated miR-223-3p upregulation via binding to the promoter of primary miR-223-3p and increasing its expression.

### miR-223-3p promotes the proliferation and migration of gastric cancer cells and is involved in CagA-mediated biological effects

**Fig. 3:**
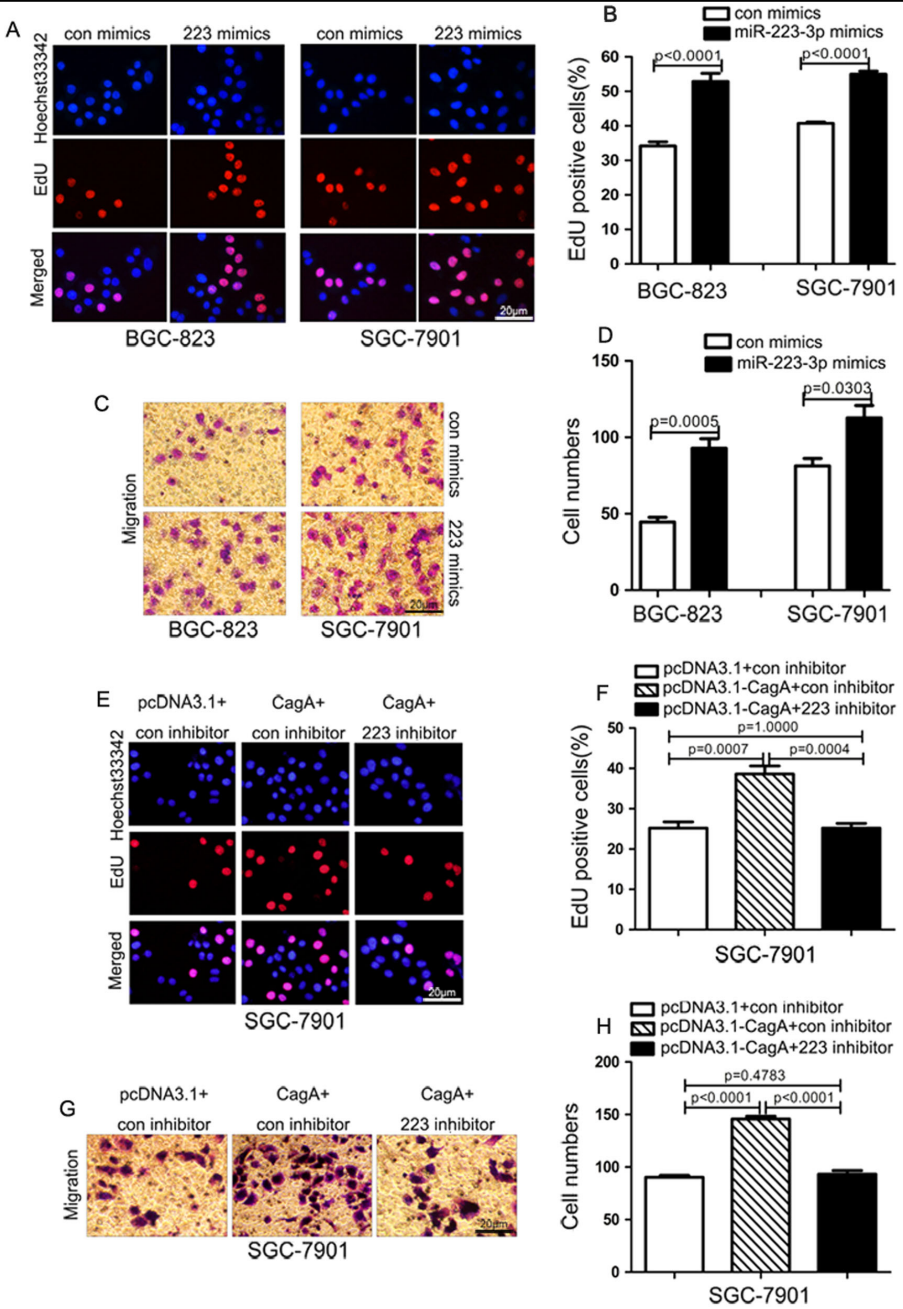
miR-223-3p promotes the proliferation and migration of gastric cancer cells and participates in CagA-mediated biological effects **a** EdU analysis of the cell proliferation ability in BGC-823 and SGC-7901 cells transfected with the control or miR-223-3p mimics. Scale bar: 20 μm. **b** Statistical analysis of the EdU-positive cell ratio in the cells transfected with the control or miR-223-3p mimics. Data are the means±SD of three independent experiments. **c** Transwell assay of the migration ability in BGC-823 and SGC-7901 cells transfected with the control or miR-223-3p mimics. Scale bar: 20 μm. **d** Statistical analysis of the cell numbers through the chamber in the transfected gastric cancer cells. Data are the means±SD of three independent experiments. **e** EdU analysis of the cell proliferation ability in SGC-7901 transfected with CagA expression vector (pcDNA3.1-CagA) alone or together with miR-223-3p inhibitor. Scale bar: 20 μm. **f** Statistical analysis of the EdU-positive cell ratio in SGC-7901 with different transfection. Data are the means±SD of three independent experiments. **g** Transwell assay of the migration ability in SGC-7901 cells transfected with CagA expression vector (pcDNA3.1-CagA) alone or together with miR-223-3p inhibitor. Scale bar: 20 μm. **h** Statistical analysis of the cell numbers through the chamber in SGC-7901 cells with different transfection. Data are the means±SD of three independent experiments

Next, we sought to elucidate the biological role of miR-223-3p in gastric carcinogenesis and progression. 5-Ethynyl-2'-deoxyuridine (EdU) and Transwell assays were used to detect the effects of the miR-223-3p mimics on gastric cancer cells proliferation and migration, respectively. As shown in Fig. 3a–d, miR-223-3p mimics promoted the proliferation and migration significantly in BGC-823 and SGC-7901 cells.We then examined whether miR-223-3p was involved in CagA-mediated biological effects. We knocked down miR-223-3p in CagA overexpressed gastric cancer cells and used EdU and Transwell assays to investigate the cell proliferation and migration ability. The results showed that *H. pylori* CagA overexpression increased the cell proliferation and migration and the miR-223-3p inhibitor partially abrogated CagA-mediated biological effects (Fig. 3e–h). Collectively, these results demonstrate that miR-223-3p plays an important role in promoting the gastric carcinogenesis and progression and is a key mediator for the biological effects of *H. pylori* CagA.

### miR-223-3p directly targets ARID1A 3’-UTR and decreases ARID1A expression

**Fig. 4:**
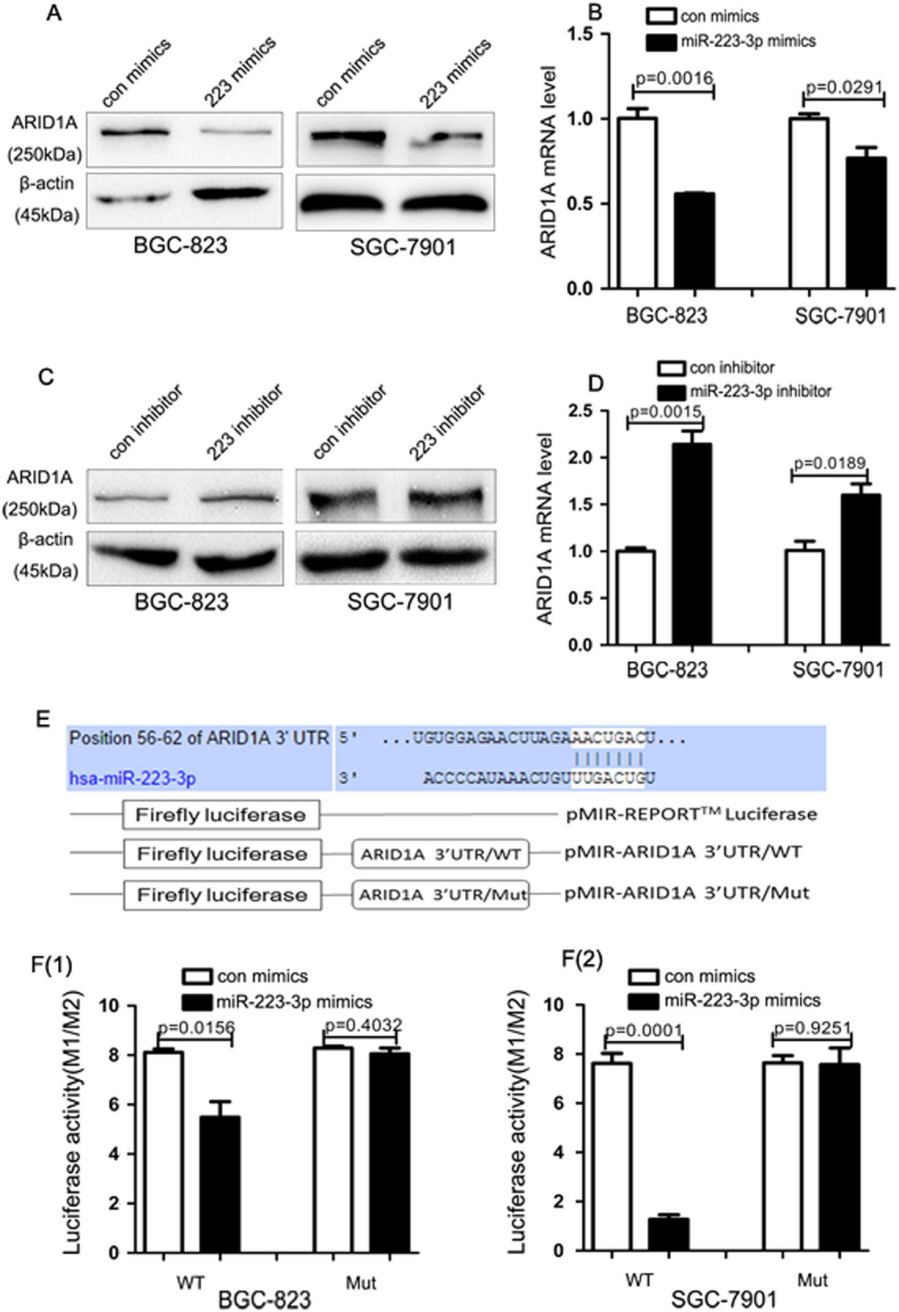
miR-223-3p downregulated ARID1A expression by directly binding to the 3’-UTR of ARID1A **a** Western blot analysis of the ARID1A protein level in BGC-823 and SGC-7901 cells transfected with control or miR-223-3p mimics. **b** qRT-PCR analysis of the ARID1A mRNA level in BGC-823 and SGC-7901 cells transfected with control or miR-223-3p mimics. **c** Western blot analysis of the ARID1A protein level in BGC-823 and SGC-7901 cells transfected with control or miR-223-3p inhibitor. **d** qRT-PCR analysis of the ARID1A mRNA level in BGC-823 and SGC-7901 cells transfected with control or miR-223-3p inhibitor. **e** Upper: the predicted complementary sequences of miR-223-3p in the 3′-UTR of ARID1A. Lower: diagram of the luciferase reporter construct containing the ARID1A 3′-UTR. The mutations were generated at the predicted miR-223-3p binding sites located in the ARID1A 3′-UTR. **f** (1 and 2) The wild-type (WT) or mutant (Mut) reporter constructs was co-transfected with control or miR-223-3p mimics into BGC-823 and SGC-7901 cells, and the dual luciferase activity was determined at 48 h after transfection

To further investigate the underlying mechanism of miR-223-3p in gastric cancer cell proliferation and migration, we used different databases such as TargetScan, Mirnada and miRBase to predict the targets of miR-223-3p and found that ARID1A (a member of the SWI/SNF family) might be targeted by miR-223-3p. Therefore, we transfected the miR-223-3p mimics or inhibitor into gastric cancer cells and used qRT-PCR and western blot to determine the effect of miR-223-3p on the expression of ARID1A. The results showed that the mRNA and protein levels of ARID1A were decreased after the ectopic expression of miR-223-3p in BGC-823 and SGC-7901 cells (Fig. 4a, b, Fig. S2). On the contrary, knockdown of miR-223-3p with miR-223-3p inhibitor increased the mRNA and protein levels of ARID1A (Fig. 4c, d, Fig. S2).

Furthermore, to ascertain whether miR-223-3p could directly target the 3′-untranslated region (UTR) of ARID1A in gastric cancer cells, we constructed a wild-type luciferase reporter vector (WT) containing the targeting sequences of ARID1A 3′-UTR and a mutant luciferase reporter vector (Mut) in which the targeting sequences were mutated (Fig. 4e). BGC-823 and SGC-7901 cells were co-transfected with WT or Mut luciferase reporters and miR-223-3p mimics. As shown in Fig. 4f, miR-223-3p overexpression suppressed the wild-type luciferase activity of ARID1A 3′-UTR reporter, while it had no effect on the Mut luciferase reporter activities. These results suggested that ARID1A was a direct target of miR-223-3p in gastric cancer cells.

### ARID1A is functionally important for the biological effects of miR-223-3p by targeting p21 and E-cadherin

**Fig. 5:**
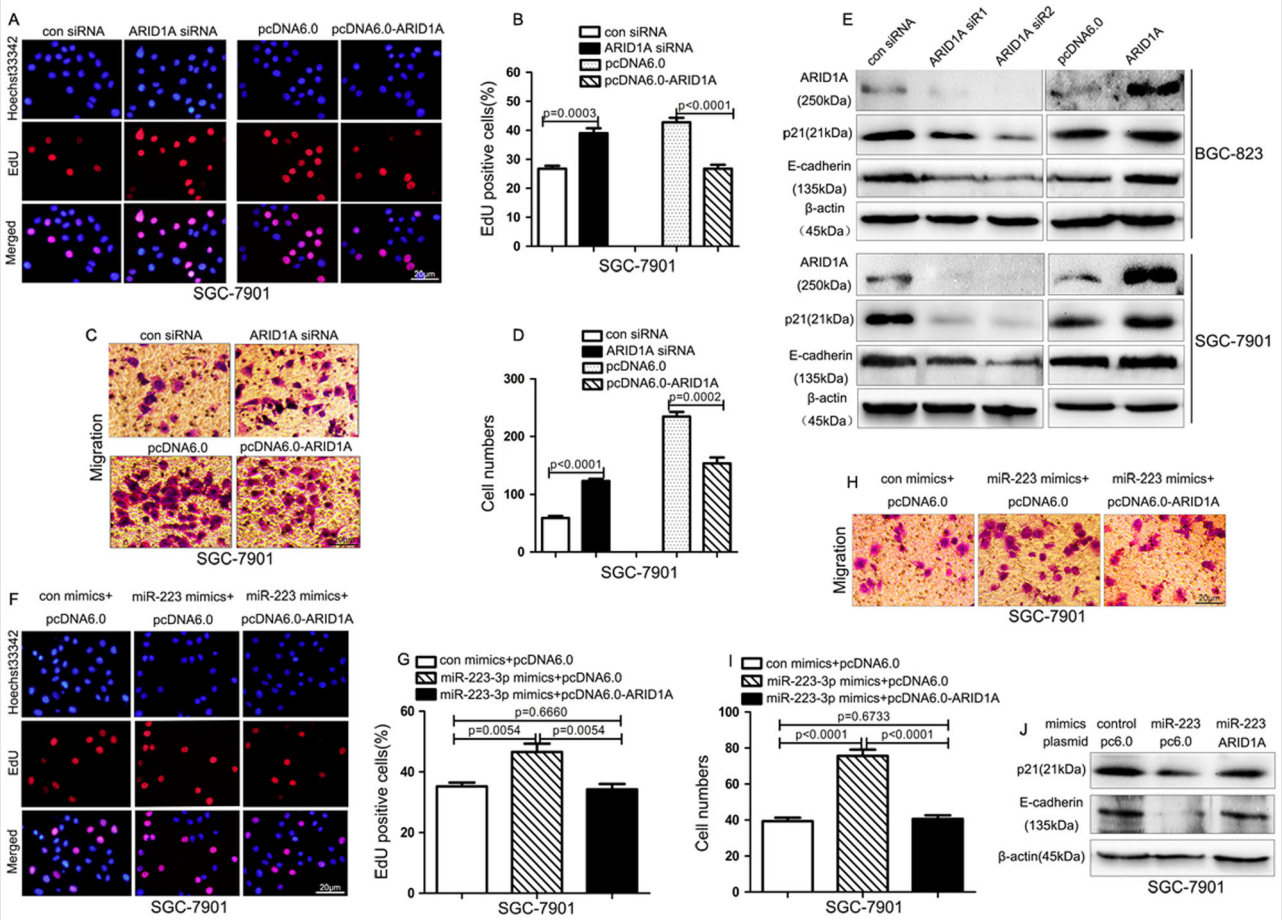
miR-223-3p promotes cell proliferation and migration by targeting ARID1A **a** EdU analysis of the cell proliferation ability in SGC-7901 cells transfected with the ARID1A siRNA or ARID1A expression vector (pcDNA6.0-ARID1A). Scale bar: 20 μm. **b** Statistical analysis of the EdU-positive cell ratio in SGC-7901 cells transfected with the ARID1A siRNA or ARID1A expression vector (pcDNA6.0-ARID1A). Data are the means±SD of three independent experiments. **c** Transwell assay of the migration ability in SGC-7901 cells transfected with the ARID1A siRNA or ARID1A expression vector (pcDNA6.0-ARID1A). Scale bar: 20 μm. **d** Statistical analysis of the cell numbers through the chamber in SGC-7901 cells transfected with the ARID1A siRNA or ARID1A expression vector (pcDNA6.0-ARID1A). Data are the means±SD of three independent experiments. **e** Western blot analysis of the expression of ARID1A, p21 and E-cadherin in SGC-7901 cells transfected with ARID1A siRNA or ARID1A expression vector (pcDNA6.0-ARID1A). **f** EdU analysis of the cell proliferation ability in SGC-7901 cells transfected with miR-223-3p mimics alone or together with ARID1A expression vector (pcDNA6.0-ARID1A). Scale bar: 20 μm. **g** Statistical analysis of the EdU-positive cell ratio in SGC-7901 cells with different transfection. Data are the means±SD of three independent experiments. **h** Transwell assay of the migration ability in SGC-7901 cells transfected with miR-223-3p mimics alone or together with ARID1A expression vector (pcDNA6.0-ARID1A). Scale bar: 20 μm. **i** Statistical analysis of the cell numbers through the chamber in SGC-7901 cells with different transfection. Data are the means±SD of three independent experiments. **j** Western blot analysis of the expression of p21 and E-cadherin in SGC-7901 cells with different transfections

We further investigated the biological role of ARID1A in GC cells and whether ARID1A was involved in miR-223-3p-mediated biological effects. We transfected ARID1A-specific siRNA or ARID1A expression vector (pcDNA6.0-ARID1A) into the gastric cancer cells and detected the effect of ARID1A on cell proliferation and migration. As shown in Fig. 5a–d, ARID1A silence promoted cell proliferation and migration, while ARID1A overexpression inhibited the proliferation and migration ability. It has been reported that cyclin-dependent kinase inhibitor p21 is a downstream target of ARID1A in gynecologic cancers and ARID1A regulates gastric cancer cell migration and invasion via regulating E-cadherin expression^25,26^. Therefore, we detected the protein levels of p21 and E-cadherin in the gastric cancer cells transfected with ARID1A siRNAs or pcDNA6.0-ARID1A. The results displayed in Fig. 5e showed that ARID1A siRNAs decreased the expression of E-cadherin and p21, while ARID1A overexpression increased the expression of E-cadherin and p21, which was consistent with previous reports.

Then, we sought to explore the potential role of ARID1A in miR-223-3p-mediated biological functions. We upregulated ARID1A expression in the cells transfected with miR-223-3p mimics and found that the increase in cell proliferation and migration mediated by the miR-223-3p mimics was abrogated by the ARID1A overexpression (Fig. 5f–i). Western blot results showed that the downregulation of p21 and E-cadherin mediated by miR-223-3p mimics can be partially reversed by the ARID1A overexpression (Fig. 5j). Therefore, these data demonstrated that miR-223-3p promoted cell proliferation and migration by targeting ARID1A partially.

### The expression of miR-223-3p is upregulated in human gastric cancer tissues and associated with *H. pylori* infection

**Fig. 6:**
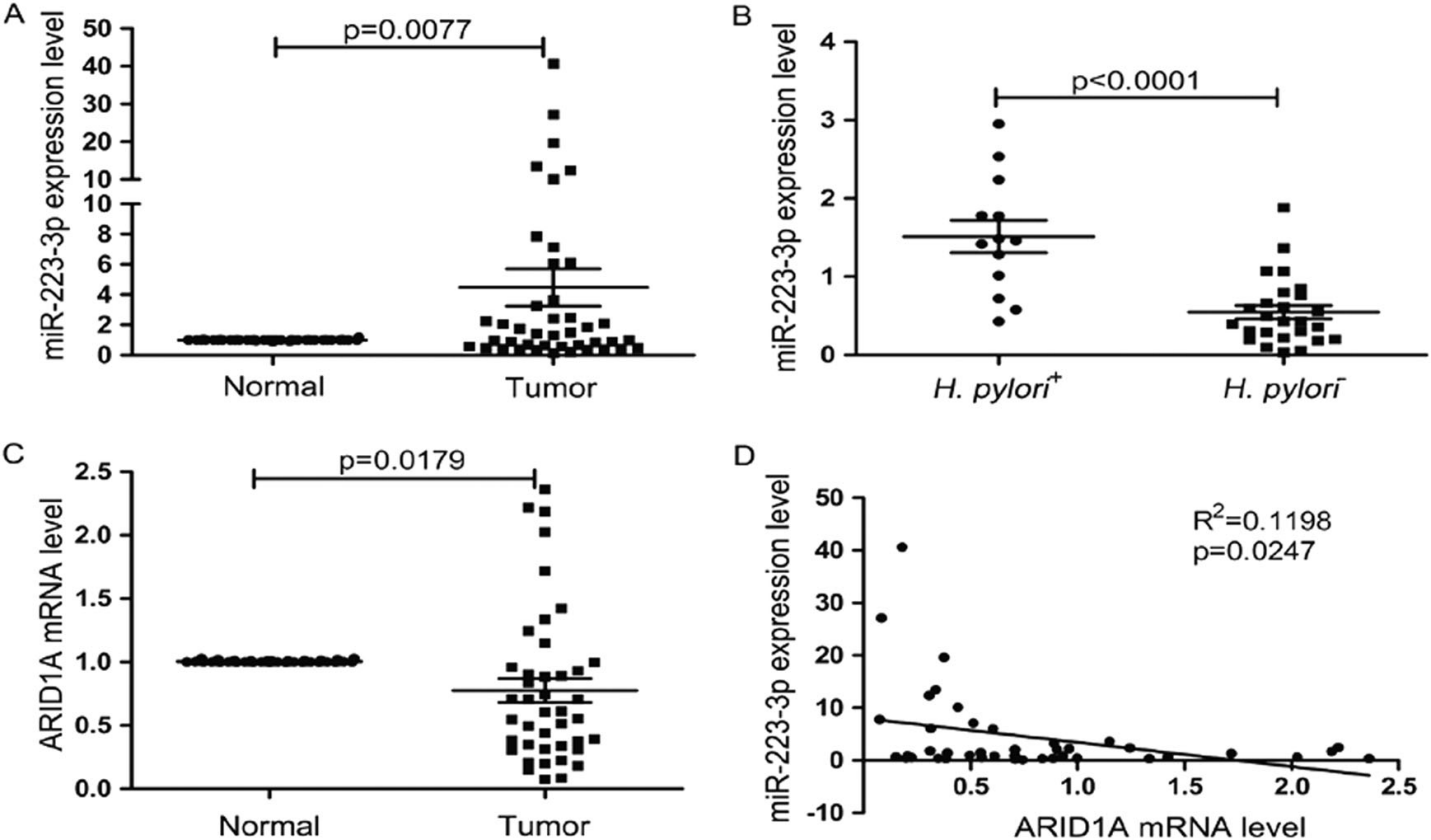
Clinical relevance of miR-223-3p and expression of ARID1A in human GC tissues. **a** Statistical analysis of miR-223-3p expression in GC tissues and adjacent normal gastric mucosa (*n* = 42, *P* = 0.0077). Horizontal lines represent the means±SD. **b** Statistical analysis of miR-223-3p expression in *H. pylori*-positive and *H. pylori*-negative gastric cancer tissues. **c** Statistical analysis of ARID1A mRNA level in GC tissues and adjacent normal gastric mucosa (*n* = 42, *P* = 0.0179). Horizontal lines represent the means±SD. **d** Regression analysis showed that ARID1A is negatively associated with miR-223-3p in GC tissues

Finally, we determined whether the results from the cell level had any clinical significance. We used qRT-PCR to detect the expression of miR-223-3p in 42 paired gastric cancer and corresponding noncancerous tissues and found that 22/42 (52.38%) of the clinical gastric cancer specimens showed significantly increased expression of miR-223-3p as compared with corresponding noncancerous tissues (Fig. S3, Table S2). Statistical analysis showed that the average expression level of miR-223-3p in tumor tissues was significantly higher than that in surrounding noncancerous tissues (Fig. 6a). Furthermore, compared with *H. pylori*-negative gastric cancer tissues, miR-223-3p expression was significantly higher in *H. pylori*-positive tissues (Fig. 6b).

### The expression of ARID1A is downregulated in human gastric cancer tissues and negatively associated with miR-223-3p

We also determined the expression of ARID1A in the paired gastric cancer and corresponding noncancerous tissues and found that 28/42 (66.7%) of the gastric cancer specimens showed significantly decreased expression of ARID1A as compared with corresponding noncancerous tissues (Fig. S4, Table S2). Statistical analysis showed that the average expression level of ARID1A in tumor tissues was significantly lower than that in surrounding noncancerous tissues (Fig. 6c). Moreover, a highly significant negative correlation between miR-223-3p and ARID1A transcripts was observed in these samples (Fig. 6d).

## Discussion

*H. pylori* infection and the resulting chronic inflammation is the leading factor for the development of gastric cancer^2,3^. Patients with *H. pylori* infection have a higher risk of gastric cancer than the *H. pylori*-negative patients. The pathogenicity of *H. pylori* is largely attributed to its various virulence components, such as flagella, lipopolysaccharide, vacuolating toxin VacA and cytotoxin-associated gene pathogenicity island (cagPAI)^27,28^. Among these, cagPAI is the most important and the most extensively studied virulence component. *H. pylori* *cagPAI* gene encoded virulence factor CagA and a T4SS. CagA protein can be injected into the gastric epithelial cells via T4SS and plays an important role in *H. pylori*-induced inflammation and tumorigenesis. It has been known that the CagA protein is required for the activation of transcription factor NF-κB^10,29–31^. NF-κB is a crucial mediator of inflammation-induced tumor growth which has become a new hallmark for cancer^32^. In addition, *H. pylori*-induced alteration of miRNA is also involved in the process of chronic inflammation to carcinogenesis^19,20^. The upregulation of miR-223-3p expression in *H. pylori*-infected gastric mucosa was detected by miRNA microarrays^21^. Moreover, Ma et al.^33^ found that the overexpression of miR-223 was related with *H. pylori*-positive infection in gastric cancer. However, the potential mechanism of *H. pylori*-mediated miR-223-3p upregulation remains undefined. Since a conserved putative NF-κB binding site in the promoter region of miR-223-3p is found^22^, we wonder whether miR-223-3p is involved in *H. pylori* CagA-mediated transformation from chronic inflammation to gastric cancer via NF-κB-dependent pathway.

In this study, we first confirmed that *H. pylori* (CagA^+^) infection increased the expression of miR-223-3p in gastric cancer cells, while CagA-deleted *H. pylori* mutant strain (CagA^-^) infection has no effect on miR-223-3p expression. Furthermore, CagA expression vector (pcDNA3.1-CagA) transfection increased the expression of miR-223-3p in the gastric cancer cells. These results suggested that *H. pylori* induced miR-223-3p expression in a CagA-dependent manner. To further explore whether NF-κB was involved in *H. pylori*-mediated upregulation of miR-223-3p, we used a specific NF-κB signal inhibitor BAY 11-7082 to treat the cells and found that NF-κB inhibitor treatment reversed *H. pylori* (CagA^+^) infection or CagA expression vector transfection-mediated upregulation of miR-223-3p. In addition, knockdown of NF-κB with specific NF-κB siRNA decreased the expression of miR-223-3p and the promoter activity of miR-223-3p. Therefore, these results suggested that *H. pylori* CagA increased miR-223-3p expression via NF-κB-dependent pathway and miR-223-3p might act as a 'bridge' to link *H. pylori*-induced chronic inflammation and carcinogenesis. Since it has been reported that miR-223 targets NF-κB and IRAK1 in macrophages^34^,we want to know whether miR-223 can target NF-κB in gastric cancer cells and form a feedback loop with NF-κB. We determined the expression of NF-κB in miR-223 mimics-transfected gastric cancer cells and found that miR-223 has no effect on the expression of NF-кB in the gastric cancer cells. We speculate that the regulation mechanism may be different in the two kinds of cells.

Next, we wondered whether miR-223-3p was involved in CagA-mediated biological role in gastric cancer. It is well known that miRNAs can regulate cell proliferation, invasion and metastasis by altering the expression of tumor suppressor genes or the oncogenes, thus contributing to the initiation and development of human tumors. miR-223-3p has been characterized as a oncogene in multiple tumors, such as pancreatic cancer, prostate cancer, ovarian cancer, lung cancer and gastric cancer, by targeting different genes, such as *FBXW7, SEPT6, SOX11, EPB41L3*^35–38^. In our study, we verified the oncogenic role of miR-223-3p in gastric cancer, which is consistent with the results from Ma et al.^33^ Importantly, we found that the miR-223-3p inhibitor reversed CagA-mediated promotion of cell proliferation and migration. In the clinical setting, miR-223-3p was downregulated in gastric cancer tissues compared with the corresponding noncancerous tissues and the expression level of miR-223-3p was significantly higher in *H. pylori*-positive gastric cancer tissues than that in *H. pylori-*negative tissues. These results suggest that miR-223-3p is involved in the CagA-mediated biological function in gastric cancer.

It is well known that miRNAs exert the biological role by targeting the protein-encoding genes and regulating the expression of these genes. In this study, we identified ARID1A as a novel direct target of miR-223-3p in gastric cancer. We found that miR-223-3p bound to the complementary sites of the 3′-UTR of ARID1A and decreased its mRNA and protein levels. ARID1A (also known as B120 and BAF250a) belongs to a family of 15 proteins in humans that all contain a characteristic 100-amino acid DNA binding ARID domain^39^. As a member of SWI/SNF complexes, ARID1A is thought to have ATPase activities and regulate transcription of certain genes by altering the chromatin structure around those genes. The tumor suppressor role of ARID1A had been identified in a wide variety of cancers, such as gastric cancer^40^, hepatocellular carcinoma^41^, breast cancer^42^ and pancreatic cancer^43^. It was noteworthy that the function of ARID1A was relevant to two processes of tumor development: proliferation and migration. In gynecological cancer, ARID1A cooperated with p53 to regulate the proliferation of tumor cells by modulating p21 and SMAD3^25^. In gastric cancer, ARID1A silencing enhanced the migration and invasion of gastric cancer cell lines via downregulation of E-cadherin expression^26^. In this report, we verified the biological role of ARID1A in gastric cancer and found that ARID1A exerted the tumor suppressor role via regulating p21 and E-cadherin. Furthermore, the overexpression of ARID1A deprived the miR-223-3p-mediated promotion of gastric cancer cell proliferation and migration, suggesting that the miR-223-3p played the oncogenic role in gastric cancer by directly targeting ARID1A.

**Fig. 7:**
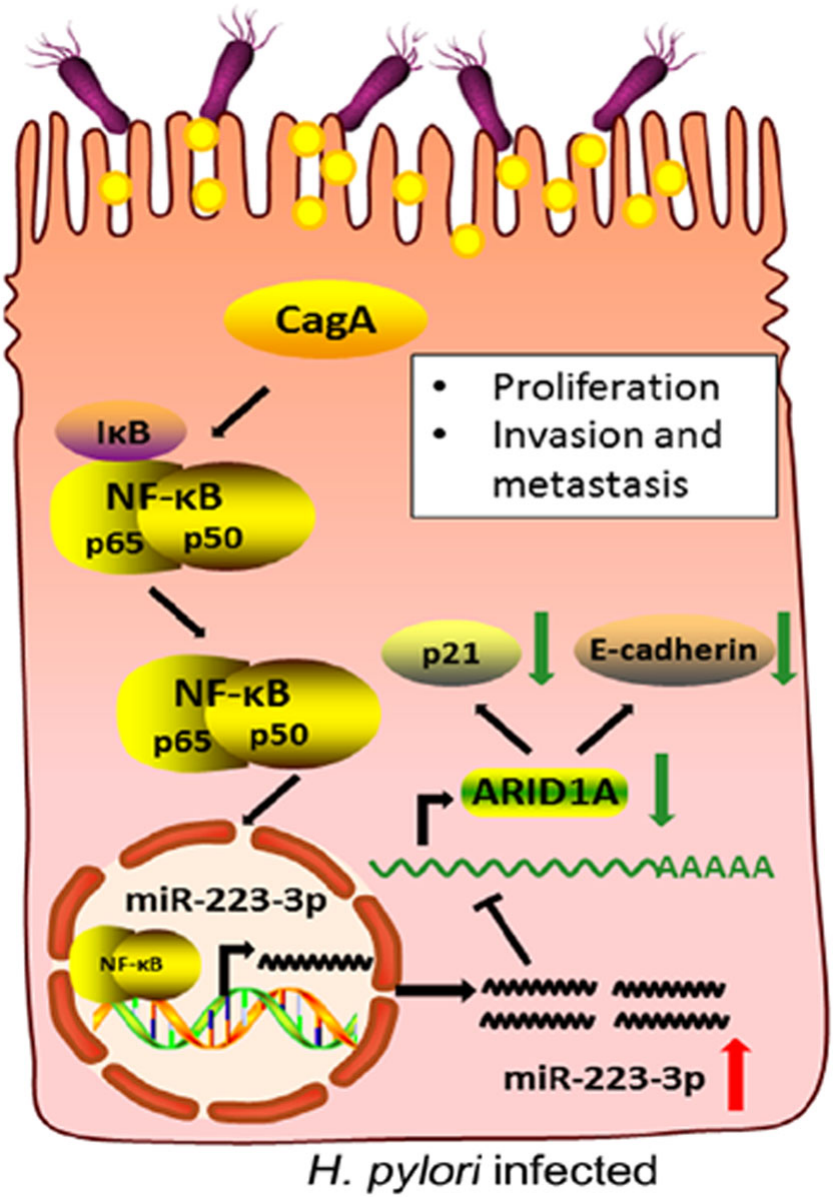
Schematic representation of the NF-κB/miR-223-3p/ARID1A regulatory mechanism in *H. pylori*-infected gastric cells

In summary, we determined the role and regulatory mechanism of miR-223-3p in *H. pylori* CagA-induced gastric carcinogenesis and progression. We found that the NF-κB/ miR-223-3p/ARID1A signaling cascade may be a 'bridge' for *H. pylori*-induced chronic inflammation to gastric cancer, thereby providing a new mechanism for the pathogenicity of *H. pylori* (Fig. 7).

## Materials and Methods

### Cell culture

Human gastric cancer cell lines AGS, BGC-823 and SGC-7901 were purchased from the Cell Resource Center, Institute of Biochemistry and Cell Biology at the Chinese Academy of Science (Shanghai, China). AGS cells were cultured in F12 medium (Invitrogen, Carlsbad, CA, USA) supplemented with 10% fetal bovine serum (FBS), 100 U/ml penicillin and 100 μg/ml streptomycin, BGC-823 and SGC-7901 cells were maintained in RPMI-1640 medium (Invitrogen) with the same supplements. All cells were cultured at 37 °C with 5% CO_2_.

### miRNAs, siRNAs and plasmid constructs

Human miR-223-3p mimics, miR-223-3p inhibitor and corresponding controls were synthesized from RiboBio (Guangzhou, China). Chemically modified Stealth siRNAs targeting NF-κB p65 (RELA), ARID1A and control siRNA were purchased from RiboBio. ARID1A eukaryotic expression vector (pcDNA6.0-ARID1A) was kindly provided by Professor Ie-Ming Shih (Departments of Pathology and Oncology and Gynecology and Obstetrics, Johns Hopkins University School of Medicine). pcDNA3.1-CagA plasmid was kindly provided by Yongliang Zhu (Zhejiang University, China). The pGL3-miR-223-3p luciferase reporter plasmid was constructed by inserting the promoter sequences of miR-223-3p into the pGL3-basic vector (Promega) between the *Kpn*I and *Xho*I sites. One assumed RELA binding site was mutated using the QuickChange Site-Directed Mutagenesis Kit and the wild-type pGL3-miR-223-3p as the template. The 3′-UTR fragments of ARID1A containing putative miR-223-3p binding sites were amplified and cloned into the pMIR-REPORT luciferase vector between *Hin*dIII and *Spe*I sites (Ambion, Austin, TX, USA). The miR-223-3p complementary sequences AACUGAC in the 3′-UTR of ARID1A were mutated to remove the complementarity. All of the constructs were verified by sequencing. All primer and siRNA sequences used in this study are listed in Table S1.

### Cell transfection

X-tremeGENE HP Transfection Reagent (Roche Applied Science) was used for the transfection of the plasmids into the gastric cancer cells (90–95% confluence). Lipofectamine 2000 (Invitrogen) was used to transfect siRNA, miRNA mimics or miRNA inhibitor into the gastric cancer cells (30–40% confluence). All of the transfection procedures followed the protocol of the manufacturer.

### *H. pylori* strain and bacterial infection

The standard *H. pylori* strain 26695 (CagA^+^) was kindly provided by Dr. Jianzhong Zhang (Chinese Disease Control and Prevention Center, Beijing, China). The isogenic 26695 *CagA* mutant strain (CagA^-^) was constructed using strain 26695 by insertional mutagenesis. The *H. pylori* strains (both CagA^+^ and CagA^-^) were inoculated into Brucella broth containing 5% FBS under microaerophilic conditions (5% O_2_, 10% CO_2_ and 85% N_2_) at 37 °C. For *H. pylori* infection, AGS, BGC-823 and SGC-7901 cells were seeded in 6-well plates and cultured to reach 80–90% confluency with antibiotics-free cell culture medium, then the *H.* *pylori* was harvested, resuspended with phosphate-buffered saline (PBS) and added to the gastric cancer cells at a multiplicity of infection of 100:1. The *H. pylori*-infected gastric cancer cells were incubated for 6 or 24 h and collected. The NF-κB inhibitor BAY 11-7082 (Sigma-Aldrich) was dissolved in dimethyl sulfoxide (Sigma-Aldrich) at 5 μmol/l and added to the cells 30 min before infection.

### RNA extraction, reverse transcription and qRT-PCR

TRIzol reagent (Invitrogen) was used to extract total RNA from the cells and tissue specimens. Primers for miR-223-3p and U6 were synthesized from RiboBio. The PCR primers for ARID1A, NF-κB p65 (RELA) and β-microglobulin (β2-M) were synthesized from the Beijing Genomics Institute. The primer sequences are listed in Table S1. The first-strand complementary DNA (cDNA) was synthesized with random primers or miRNA-specific primers and RevertAid First Strand cDNA Synthesis kit (Thermo Scientific™). Then, qRT-PCR was performed using the Bio-Rad CFX96TM Real-Time PCR System (Bio-Rad) with the SYBR Green Kit (TaKaRa) according to the manufacturer’s instructions. Calculation of the target mRNA or miRNA levels was based on the 2^-ΔΔCt^ method and normalization to human β2-M or U6 expression. All of the reactions were run in triplicate.

### Western blot analysis

Total proteins from the cells or tissues were extracted with RIPA lysis buffer containing proteinase inhibitor. The protein concentrations were measured by the BCA reagent kit (Beyotime). The proteins were separated by sodium dodecyl sulfate–polyacrylamide gel electrophoresis and transferred to polyvinylidene difluoride membranes, which were blocked in 5% non-fat milk, and then incubated with the primary antibodies against ARID1A (Bethyl Laboratories), NF-κB p65 (RELA) (Santa Cruz), E-cadherin (Santa Cruz), CagA (Santa Cruz), p21 (Proteintech) and β-actin (Abcam) at 4 °C overnight. The membranes were then washed in tris buffered saline tween and incubated with anti-mouse or rabbit horseradish peroxidase-conjugated secondary antibodies and developed with the enhanced chemiluminescence method (ECL, Millpore). β-actin served as a loading control.

### Luciferase reporter assay

Gastric cancer cells were seeded in 24-well plates (3 × 10^4^ cells/well) and were transiently transfected with the siRNA or miRNA and appropriate reporter plasmid using Lipofectamine 2000 or X-tremeGENE HP Transfection Reagent. After 48 h, the cells were harvested and lysed in passive lysis buffer. Luciferase activity was measured using the Dual-Luciferase Reporter Assay System (Promega, Madison, WI, USA) according to the manufacturer’s protocol. Renilla luciferase was used for normalization. The transfection experiments were performed in triplicate.

### Transwell assay

Gastric cancer cells with different transfection treatments were harvested and resuspended in serum-free RPMI-1640 medium, and 1 × 10^5^ cells were seeded into the upper 24-well chambers. RPMI-1640 medium containing 20% FBS was added to the lower chambers as a chemoattractant. After 24 h, cells remaining on the upper surface of the membrane were removed with a cotton swab, and cells that had migrated the membrane filter were fixed with 100% methanol, stained in a dye solution containing 0.05% crystal violet and photographed under a microscope. The number of migration cells was counted from three independent experiments.

### EdU assay

Cell proliferation assay was performed using the Cell-Light™ EdU Apollo®567 In Vitro Imaging Kit (RiboBio Co.). Briefly, at 48 h after transfection, the cells were seeded in 96-well plates at a density of 8 × 10^3^cells/well. Additionally, the cells were incubated with 50 μmol/l EdU for 2 h at 37 °C. After being fixed with 4% paraformaldehyde for 30 min, the cells were treated with 0.5% Triton X-100 for 10 min and rinsed with PBS three times. Thereafter, the cells were exposed to 100 μl of 1 × Apollo® reaction cocktail for 30 min and then incubated with 5 μg/ml of Hoechst 33342 to stain the cell nuclei for 30 min. Images were captured using a fluorescent microscope (Olympus, Tokyo, Japan). The percentage of EdU-positive cells was defined as the proliferation rate. All of the experiments were performed in triplicate.

### Patients

We obtained fresh tumor specimens and surrounding normal tissue from patients with primary gastric cancer who underwent gastrectomy at the Center Hospital of Taian City in 2016–2017. Samples were stored at −80 °C. We collected data on patient age, sex, tumor histology, differentiation status, size (diameter), invasiveness and regional and distant metastases at the time of surgery (pathologic tumor–node–metastasis classification) in Table S2. The study was approved by the ethics committee of School of Medicine, Shandong University.

### Statistical analysis

Comparisons between different groups were analyzed by Student’s *t*-test. Correlation analysis between ARID1A and miR-223-3p expression in GC samples were made using linear regression. Statistical analyses were performed with the Statistical Package for the Social Sciences, version 17.0 (SPSS Inc., Chicago, IL, USA), and *P* < 0.05 was considered statistically significant.

## Electronic supplementary material

TableS1

TableS2

FigureS1

FigureS2

FigureS3

FigureS4

FigureS5
