# Supplementary material for: NF-κB/miR-223-3p/ARID1A axis is involved in Helicobacter pylori CagA-induced gastric carcinogenesis and progression
Source: Cell Death Dis. 2018 Jan 9;9(1):12. doi: 10.1038/s41419-017-0020-9 (PMC5849037; doi:10.1038/s41419-017-0020-9)
Supplement: Supplementary file 1 — TableS1 [file 41419_2017_20_MOESM1_ESM.doc]

Table S1. Sequences for siRNAs and primers used in this study

| **siRNAs and primers** | **Sequences** |
| --- | --- |
| **siRNAs** |  |
| Control siRNA | 5’-CCUACAUCCCGAUCGAUGAUGUUGA-3’ |
| NF-κB p65(RELA) siRNA1 | 5’-CCAUCAACUAUGAUGAGUU-3’ |
| NF-κB p65(RELA) siRNA2 | 5’-CUUCCAAGUUCCUAUAGAA-3’ |
| Arid1a siRNA1 | 5’-GGACCUCUAUCGCCUCUAU-3’ |
| Arid1a siRNA2 | 5’-CGAGGAUGCCUGAUUGAGA-3’ |
| **Primers for promoter construct** |  |
| 223 promoter (forward) | 5’-GGTACCCAAAGTCAACTACTTTCTTCTCCCTT-3’ |
| 223 promoter (reverse) | 5’-CTCGAGCCAGATGGAATTGGGCTTT-3’ |
| mutant 223 promoter (forward) | 5’-CCCTCAATTATTCTCCCTAAACC-3’ |
| mutant223 promoter (reverse) | 5’-GGTTTAGGGAGAATAATTGAGGG-3’ |
| **primers for 3’-UTR construct** |  |
| Arid1a 3’-UTR (forward) | 5’-GGACTAGTTAGTGTGGACATGATGCGGC-3’ |
| Arid1a 3’-UTR (reverse) | 5’-CCCAAGCTTGTTATGAAATGTGGCATCCCG-3’ |
| Arid1a 3’-UTR mutant (forward) | 5’-ACTTAGATTGACGCTGTTGCC-3’ |
| Arid1a 3’-UTR mutant (reverse) | 5’-CAGCGTCAATCTAAGTTCTCC-3’ |
| **primers for real-time PCR** |  |
| β_2_-M (forward) | 5’-GAATTGCTATGTGTCTGGGT-3’ |
| β_2_-M (reverse) | 5’-CATCTTCAAACCTCCATGATG-3’ |
| Arid1a (forward) | 5’-CTTCAACCTCAGTCAGCTCCCA-3’ |
| Arid1a (reverse) | 5’-GGTCACCCACCTCATACTCCTTT-3’ |
| NF-κB p65(RELA) (forward)  NF-κB p65(RELA) (reverse) | 5’-GCCTGTCCTTTCTCATCCCA-3’  5’-CTGCCAGAGTTTCGGTTCAC-3’ |
