# Supplementary material for: NF-κB/miR-223-3p/ARID1A axis is involved in Helicobacter pylori CagA-induced gastric carcinogenesis and progression
Source: Cell Death Dis. 2018 Jan 9;9(1):12. doi: 10.1038/s41419-017-0020-9 (PMC5849037; doi:10.1038/s41419-017-0020-9)
Supplement: Supplementary file 2 — TableS2 [file 41419_2017_20_MOESM2_ESM.doc]

Table S2. Patient and tumor characteristics, miR-223-3p and ARID1A expression in normal and gastric cancer specimens

| **Patient and tumor characteristics** | | | | |  | **miR-223-3p expression**^3^ | |  | **ARID1A expression**^3^ | |
| --- | --- | --- | --- | --- | --- | --- | --- | --- | --- | --- |
| No. | Age/Sex | Size(cm) | pTNM^1^ | Histol/Dif^2^ |  | Normal | Tumor |  | Normal | Tumor |
| 1 | 49/F | 3.0 | T4N1M0 | LDA |  | 1.031 | 1.481 |  | 1.011 | 0.548 |
| 2 | 58/M | 2.0 | T4N1M0 | MDA |  | 1.005 | 0.215 |  | 0.999 | 0.709 |
| 3 | 59/M | 5.0 | T4N3M0 | LDA |  | 0.935 | 2.238 |  | 1.000 | 0.960 |
| 4 | 68/M | 12.0 | T4N2M1 | LDA |  | 1.002 | 7.849 |  | 1.006 | 0.076 |
| 5 | 60/M | 5.8 | T4N1M0 | LDA |  | 1.004 | 0.363 |  | 0.999 | 1.336 |
| 6 | 45/F | 6.0 | T4N3M0 | LDA |  | 1.005 | 12.381 |  | 1.000 | 0.308 |
| 7 | 56/F | 4.5 | T3N3M1 | LDA |  | 1.001 | 2.104 |  | 0.999 | 0.904 |
| 8 | 83/M | 7.0 | T3N0M0 | MDA |  | 0.897 | 6.045 |  | 0.998 | 0.605 |
| 9 | 52/M | 3.5 | T4N3M0 | LDA |  | 1.004 | 10.117 |  | 1.009 | 0.442 |
| 10 | 54/M | 5.5 | T4N1M0 | MDA |  | 1.004 | 0.378 |  | 1.000 | 0.385 |
| 11 | 77/M | 5.5 | T4N3M1 | L-MDA |  | 1.008 | 0.687 |  | 1.002 | 1.422 |
| 12 | 56/M | 7.5 | T4N3M0 | LDA |  | 1.005 | 3.250 |  | 0.999 | 0.891 |
| 13 | 69/M | 1.0 | T1N0M0 | HDA |  | 1.003 | 7.138 |  | 1.020 | 0.514 |
| 14 | 65/M | 3.0 | T4N1M0 | MDA |  | 1.004 | 0.352 |  | 1.021 | 2.361 |
| 15 | 51/M | 10.0 | T4N3M1 | MDA |  | 1.003 | 3.648 |  | 0.998 | 1.150 |
| 16 | 51/M | 3.5 | T4N2M0 | LDA |  | 1.013 | 1.841 |  | 1.005 | 0.312 |
| 17 | 72/M | 10.5 | T4N0M0 | L-MDA |  | 1.051 | 0.664 |  | 1.002 | 0.151 |
| 18 | 77/M | 14.0 | T4N3M1 | LDA |  | 1.175 | 0.873 |  | 0.999 | 0.708 |
| 19 | 53/F | 6.0 | T4N3M0 | LDA |  | 1.020 | 6.143 |  | 1.002 | 0.314 |
| 20 | 69/M | 7.0 | T4N3M0 | LDA |  | 1.005 | 0.460 |  | 1.001 | 0.200 |
| 21 | 69/M | 3.0 | T4N2M0 | LDA |  | 1.001 | 0.414 |  | 1.008 | 0.885 |
| 22 | 82/M | 1.0 | T2N0M0 | LDA |  | 1.032 | 0.133 |  | 1.009 | 0.743 |
| 23 | 47/M | 2.0 | T2N1M0 | LDA |  | 1.016 | 0.410 |  | 1.008 | 0.349 |
| 24 | 73/F | 8.0 | T4N3M0 | L-MDA |  | 1.000 | 13.492 |  | 0.998 | 0.338 |
| 25 | 75/M | 7.0 | T4N3M0 | LDA |  | 1.011 | 40.640 |  | 1.003 | 0.182 |
| 26 | 49/F | 8.0 | T3N3M0 | LDA |  | 1.006 | 1.746 |  | 1.012 | 2.186 |
| 27 | 79/M | 8.0 | T3N1M0 | MDA |  | 1.015 | 1.306 |  | 1.003 | 1.718 |
| 28 | 77/M | 3.0 | T1N0M0 | MDA |  | 0.963 | 2.460 |  | 1.001 | 2.218 |
| 29 | 63/M | 5.0 | T4N3M0 | MDA |  | 1.008 | 19.636 |  | 1.014 | 0.378 |
| 30 | 56/M | 5.0 | T4N3M1 | LDA |  | 1.000 | 0.869 |  | 1.002 | 0.931 |
| 31 | 60/M | 2.0 | T2N2M0 | MDA |  | 1.007 | 0.627 |  | 1.005 | 2.026 |
| 32 | 64/M | 3.0 | T4N2M0 | MDA |  | 1.009 | 0.390 |  | 1.002 | 0.835 |
| 33 | 77/M | 4.0 | T4N3M0 | L-MDA |  | 1.008 | 0.569 |  | 1.001 | 0.552 |
| 34 | 70/F | 0.9 | T2N0M0 | L-MDA |  | 1.001 | 0.974 |  | 1.001 | 0.496 |
| 35 | 48/F | 6.0 | T4N3M0 | LDA |  | 1.003 | 0.833 |  | 1.002 | 0.612 |
| 36 | 66/M | 6.0 | T4N3M0 | L-MDA |  | 1.005 | 0.572 |  | 1.029 | 0.224 |
| 37 | 65/M | 3.0 | T4N3M0 | LDA |  | 1.006 | 0.459 |  | 1.001 | 0.997 |
| 38 | 53/M | 3.5 | T2N1M0 | L-MDA |  | 1.001 | 0.949 |  | 1.001 | 0.206 |
| 39 | 77/M | 2.0 | T4N0M0 | MDA |  | 1.000 | 1.422 |  | 1.007 | 0.393 |
| 40 | 74/M | 4.0 | T2N1M0 | LDA |  | 1.001 | 27.139 |  | 1.002 | 0.084 |
| 41 | 65/M | 7.0 | T4N3M0 | LDA |  | 1.008 | 2.065 |  | 1.005 | 0.708 |
| 42 | 52/M | 2.5 | T4N3M0 | M-HDA |  | 1.003 | 2.396 |  | 1.027 | 1.245 |

1. Pathologic tumor-node-metastasis.

2. Histology/Differentiation status:

LDA, Low differentiated adenocarcinoma;

MDA, Moderately differentiated adenocarcinoma;

HAD, High differentiated adenocarcinoma.

3. qRT-PCR analysis of miR-223-3p and ARID1A expression in normal and tumor tissues.
