# Supplementary figures and images for: NF-κB/miR-223-3p/ARID1A axis is involved in Helicobacter pylori CagA-induced gastric carcinogenesis and progression
Source: Cell Death Dis. 2018 Jan 9;9(1):12. doi: 10.1038/s41419-017-0020-9 (PMC5849037; doi:10.1038/s41419-017-0020-9)

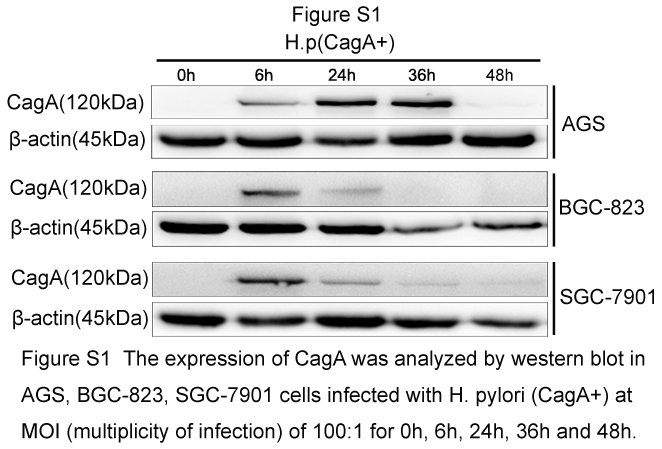

Supplement: Supplementary file 3 — FigureS1 [file 41419_2017_20_MOESM3_ESM.tif]

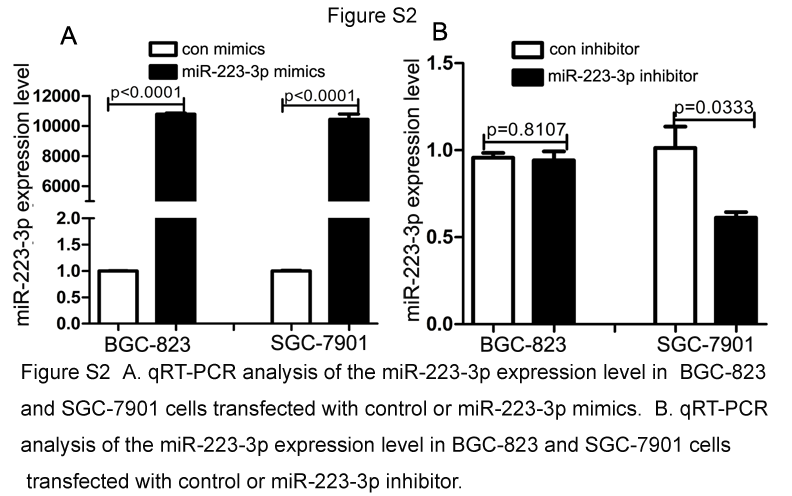

Supplement: Supplementary file 4 — FigureS2 [file 41419_2017_20_MOESM4_ESM.tif]

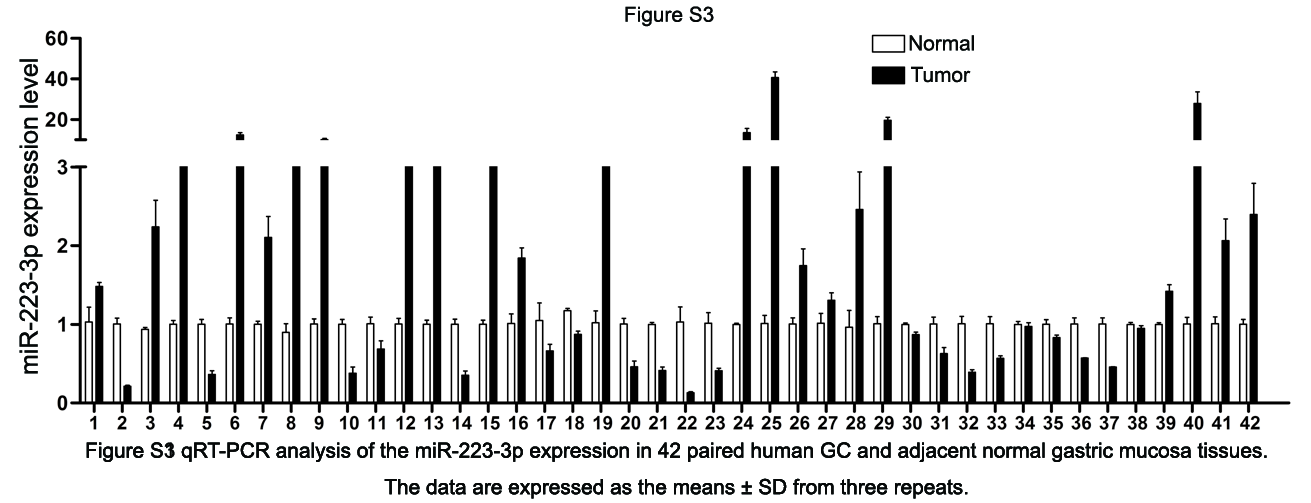

Supplement: Supplementary file 5 — FigureS3 [file 41419_2017_20_MOESM5_ESM.tif]

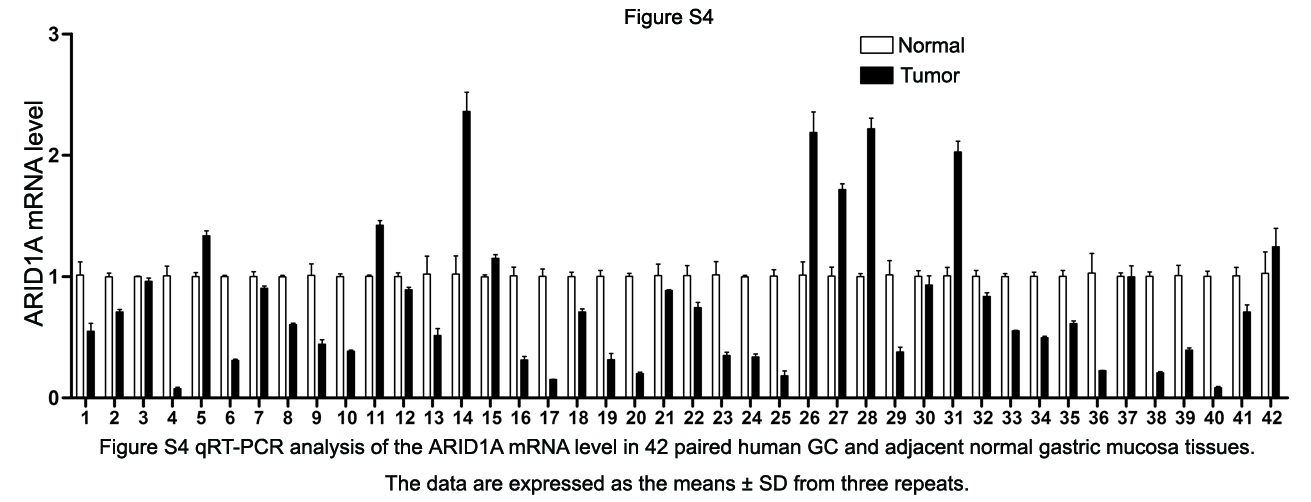

Supplement: Supplementary file 6 — FigureS4 [file 41419_2017_20_MOESM6_ESM.tif]

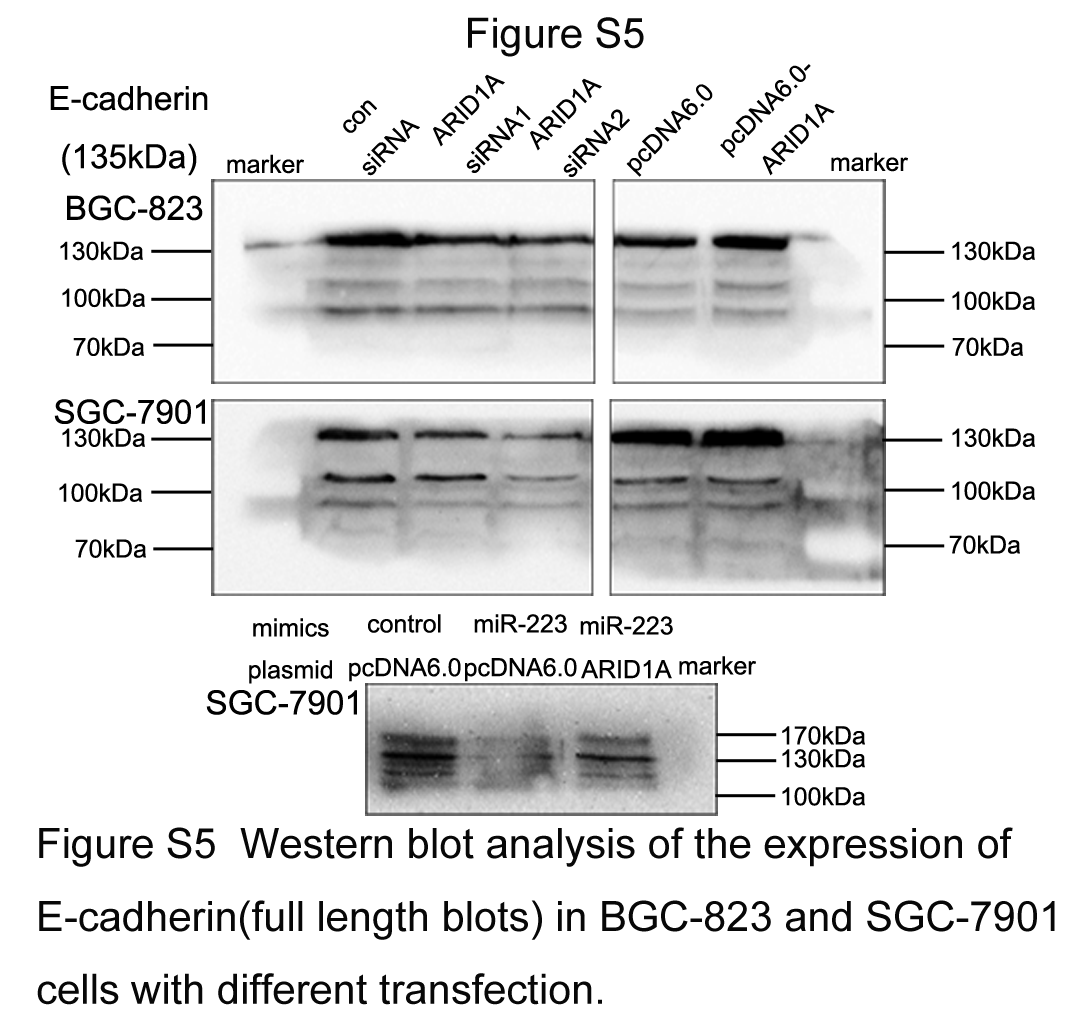

Supplement: Supplementary file 7 — FigureS5 [file 41419_2017_20_MOESM7_ESM.tif]
